# Factors in homeowners’ willingness to adopt nitrogen-reducing innovative/alternative septic systems

**DOI:** 10.3389/fmars.2023.1069599

**Published:** 2023-05-23

**Authors:** Alexie N. Rudman, Kate K. Mulvaney, Nathaniel H. Merrill, Katherine N. Canfield

**Affiliations:** 1 Office of Research and Development, United States Environmental Protection Agency, Narragansett, RI, United States; 2 Oak Ridge Institute for Science and Education, Oak Ridge Associated Universities, Oak Ridge, TN, United States

**Keywords:** best management practices (BMPs), nonpoint source pollution, sociology, estuaries, social science, technology adoption, water quality

## Abstract

When environmental mitigation requires individual adoption, engagement approaches centered on cost effectiveness and technological efficiency alone are often insufficient. Through focus groups with adopters and prospective adopters, this research identifies factors influencing homeowners’ willingness to adopt Innovative/Alternative (I/A) septic systems for nitrogen reduction. We apply concepts from technology adoption and behavior change models as a framework for illustrating the homeowner decision-making process around I/A adoption. The perceived needs to replace an old/failing septic system, comply with local regulations, and protect local water quality synergistically catalyzed adoption. Adoption was further influenced by the larger context within which it is taking place, perceived characteristics of I/A systems and the installation process, system aesthetics concerns, and homeowners’ attitudes and beliefs. Considering how these factors affect adoption could enable resource managers to design more targeted interventions to encourage adoption through behavior change strategies such as social marketing, and to improve how these systems are communicated. If I/A systems are to be used as a tool to achieve water quality goals, the considerations influencing homeowners’ willingness to install these systems will be critical to incentivizing voluntary adoption. Many of these factors are relevant to the adoption of other individual-level environmental management strategies, which are being increasingly deployed in response to complex, nonpoint source pollution issues.

## 1 Introduction

Excess nitrogen in coastal waters is responsible for widespread water quality problems on Cape Cod (Cape Cod Commission, 2015; Costa et al., 2018) and in nearly all marine estuaries (Camargo and Alonso, 2006; Howarth, 2008). As a production-limiting nutrient in marine environments, excess nitrogen inputs to coastal waters from wastewater, fertilizers, and atmospheric deposition stimulate algal growth leading to eutrophication, hypoxia, and other impairments (Howarth, 2008; Costa et al., 2018). The resulting decline in water quality translates to ecological, human health, and socioeconomic impacts (Backer and McGillicuddy, 2006; Landrigan et al., 2020; Merrill et al., 2020). On Cape Cod, onsite septic systems are the primary source of nitrogen loading, responsible for 94% of nitrogen from wastewater and 80% of the controllable nitrogen load.^1^ Water quality issues are exacerbated by a significant population influx in the summer months applying pressure to existing wastewater infrastructure, a ten to 100-year time lag in groundwater reaching estuaries, and geological characteristics such as shallow groundwater and porous sandy soils (Walter et al., 2005; Costa et al., 2018).

Although there have long been efforts to identify best management practices (BMPs) to reduce nitrogen pollution and improve water quality in the region (Cape Cod Commission, 2015; Heufelder, 2019), targeted action was recently spurred by a lawsuit filed against the United States Environmental Protection Agency (EPA) for neglecting to enforce the implementation of Cape Cod’s Area Wide Water Quality Management Plan [(Conservation Law Foundation, Inc. v. United States Environmental Protection Agency, 2013; Federal Water Pollution Control Act, 1977) 33 U.S.C. §§ 208]. As required by Section 208 of the Clean Water Act, an updated version of Cape Cod’s Area Wide Water Quality Management Plan (commonly referred to as the “208 Plan”) urges municipalities to expand nutrient reduction efforts to meet Total Maximum Daily Load limits for nitrogen inputs to coastal waters (Cape Cod Commission, 2015). It advocates for municipalities to transcend political boundaries and embrace a watershed-based approach to address nitrogen loading, emphasizing the role of traditional (sewering and wastewater treatment plants) and alternative wastewater management technologies (e.g., shellfish aquaculture or permeable reactive barriers, etc.) to address septic system effluent and other sources of nitrogen pollution (Cape Cod Commission, 2015).

**FIGURE 1:**
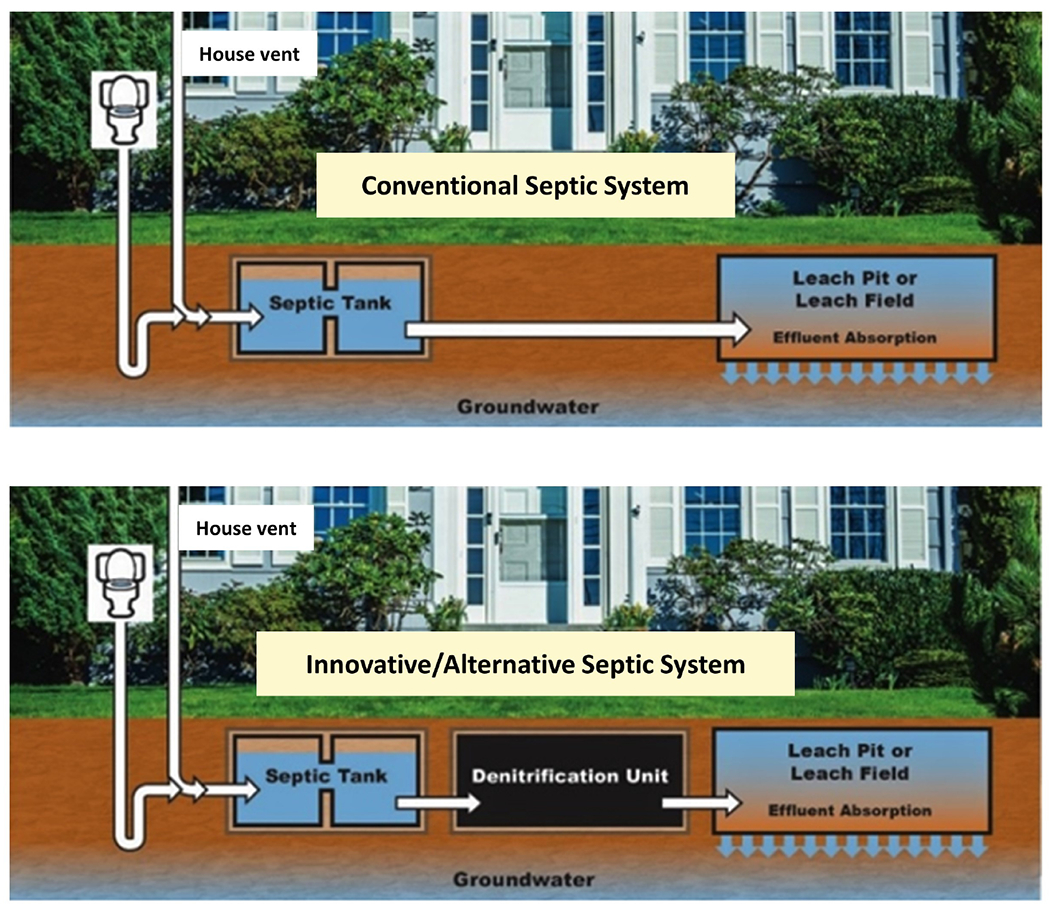
Graphic illustration of a nitrogen-reducing I/A septic system compared to a traditional septic system, modified from epa.gov.

Innovative/Alternative (I/A) septic systems (sometimes called Alternative and Experimental (A/E) technologies or nitrogen-reducing septic systems) are increasingly being considered for onsite wastewater treatment to replace traditional septic systems in nitrogen-sensitive areas. Traditional septic systems, a term used interchangeably with conventional systems and often referred to as ‘Title 5’ systems in Massachusetts, generally consist of a septic tank used to separate wastewater effluent from solids and a leaching field to treat wastewater for pathogen removal (Costa et al., 2002). I/A systems have an additional treatment component such as a peat filter, sawdust or woodchips, a recirculating sand filter, or an aerobic treatment unit to remove nitrogen from wastewater, as illustrated in Figure 1. This additional component relies on bacterial activity to first enable ammonification, then nitrification, and finally denitrification. While septic systems effectively remove most pathogens, they do not effectively remove nitrogen from wastewater before it enters groundwater (Heufelder, 2019). This inadequate treatment can jeopardize public health, groundwater, and surface waters, especially when located in nutrient-sensitive watersheds (Cape Cod Commission, 2015); Town of Eastham, 2020 I/A septic systems are specifically designed to remove nitrogen from wastewater effluent, and a newer generation of ‘enhanced I/A systems’ appears to remove nitrogen more efficiently (Heufelder, 2019; Gobler et al., 2021).

To date, I/A systems have not been widely adopted and are primarily installed as a result of local mandates or on a pilot basis^2^ (Wood et al., 2015; Buzzards Bay Coalition, 2017), except for a county-wide initiative in Suffolk County, Long Island^3^. I/A systems are sometimes mandated when conventional septic systems cannot be constructed in accordance with Massachusetts Title 5 regulations due to lot size, groundwater elevations, or nitrogen-sensitive areas (e.g., Town of Westport, 2021). Nitrogen-sensitive areas, described in the state environmental code (Title 5 of the State Environmental Code, 310 Code Mass. Regs. §§ 15.000), include wellhead protection areas near private or public wellheads, areas where an aquifer contributes water to a well, and state-designated nitrogen-sensitive embayments. Towns may identify additional nitrogen or environmentally sensitive areas such as vernal pools, ponds, and coastal beaches, and can impose more stringent restrictions based on local conditions for the installation, maintenance, and monitoring of these systems than those established by the state environmental code (Town of Dennis, 2005; Town of Eastham, 2020; Town of Marion, 2020). Local boards of health can also require I/A systems to be installed for new construction to meet local environmental limits (Town of Westport, 2021). These systems can be used by builders or homeowners to increase the volume of wastewater flow allowed in nitrogen-sensitive areas (Town of Eastham, 2020; Town of Marion, 2020), allowing for the construction of additional bedrooms beyond those already permitted in nitrogen-sensitive areas. Recent proposed changes to the state environmental code could require the installation of I/A systems in certain instances in designated Natural Resource Nitrogen Sensitive Areas, but these changes have not been finalized.^4^

Like other novel and alternative technologies, if I/A systems are to be implemented as a nutrient-mitigation tool they must be socially desirable. Facilitating adoption requires an understanding of the conditions and considerations that drive individual behavior (Steg, 2016), including but not limited to cost, access to technology, policies and regulations, local context, and attitudes and beliefs. Regional efforts have primarily focused on the economic and technical efficiency of nutrient-mitigating technologies, with little emphasis on understanding homeowner decision-making around the adoption of technologies like I/A systems (Wood et al., 2016; Heufelder, 2019; Twichell et al., 2019). The need for research on behavior change and the ‘social acceptance’ of alternative technologies in the region was identified in a regional 2019 workshop aimed at identifying knowledge gaps and needs to address Cape Cod’s nutrient loading (Twichell et al., 2019) and in prior research on designing solutions for clean water on the Cape (Perry et al., 2020). Specific research priorities include the need to understand the role of incentives and policy in motivating public participants and the identification of social, educational, and engagement barriers to adoption (Twichell et al., 2019).

Human behavior continues to contribute to environmental problems, including nutrient loading in estuaries and freshwater bodies. At an individual level, targeted shifts in human behavior are required to address environmental problems through the adoption of new ideas and technologies. To encourage behavioral change, we must understand how individuals make decisions. Influenced by contextual factors internal and external to an individual, behavior, at its most basic level, is an action that results from individual decision-making. The decision to act is roughly composed of psychological (values, attitudes, personal norms, intentions) and contextual (incentives, social norms, technologies) elements that interact and even compete to shape actions and decisions (Kollmuss and Agyeman, 2002; Wilson and Dowlatabadi, 2007; Straub, 2009).

For decades, researchers’ studies on how individuals behave and make decisions have led to different models and theories of decision-making. The most cited models are rooted in behavioral economics, technology adoption, social and environmental psychology, and sociology (Wilson and Dowlatabadi, 2007). These are intended to help researchers and policymakers understand decision-making so that interventions can be designed to account for factors that influence behavioral change. These models and theories help us understand how and why individuals make decisions, offering insights into the innumerable cognitive, social, and external contextual factors influencing decision-making (Wilson and Dowlatabadi, 2007).

In describing how innovations come about and become widely adopted, technology adoption theory posits that social processes and the characteristics of an innovation largely determine adoption (Wilson and Dowlatabadi, 2007; Straub, 2009). Technology adoption theories like the Diffusion of Innovations (DOI) consider contextual factors making up the environment and surroundings during the adoption process, unlike the Reasoned Action Approach model (RAA), as well as cognitive and affective factors like attitudes (Rogers, 2003; Straub, 2009). Rogers’ DOI establishes that social systems, innovations themselves, timing, and communication channels influence the diffusion of an innovation, defined as the process by which innovation is communicated overtime among participants of a social system through communication channels (2003). Rogers suggests that how individuals perceive characteristics of an innovation explains different rates of adoption (Rogers, 2003). The RAA focuses on internal cognitive factors driving adoption, in which attitudes towards a behavior lead to beliefs about a behavior, which interact with perceived personal and social norms to form intention (the likelihood of action), which determines behavior (Fishbein and Ajzen, 2010). The outcomes of both models are behavior change, and both are based on individuals’ perceptions or beliefs regardless of whether these perceptions are correct (Reimer et al., 2012). In Reimer et al. (2012) exploration of agricultural best management practice (BMP) adoption, the authors outlined an integrated model of the Reasoned Action Approach (Fishbein and Ajzen, 2010) with the Diffusion of Innovations (Rogers, 2003) that highlights the important connections across these theories.

Studies on the human dimensions of wastewater technology adoption in the United States are scant (Warner et al., 2022; Balkema et al., 2002; Mulvaney et al., 2023), and those focused on I/A septic systems are even more so. Only a handful of I/A septic system research projects exist within the United States, most of which are implemented at a spatially limited scale^5^ (Buzzards Bay Coalition, 2017; Mezzacapo, 2019). Few of these have considered aspects of adoption other than nitrogen-reduction efficiency and cost (Wood et al., 2016). For example, an I/A system pilot on Cape Cod identified cost as the most significant factor influencing I/A adoption, stating that participating homeowners wanted systems that were “simple, affordable, reliable, and out-of-sight” (Buzzards Bay Coalition, 2017). The same can be said about I/A system research efforts globally, which focus primarily on technical efficiency (Gill et al., 2009; Otawa and Tabata, 2014; Gunady et al., 2015) and only seldomly consider the social and cognitive aspects of adoption, apart from global studies on the social acceptance of urine-diverting toilets (Lamichhane and Babcock, 2013; Bing, 2014; Sook Eom et al., 2021). References to the social aspects of adopting these systems are surface level at best.

To understand the conditions and considerations that influence homeowners’ decisions to install I/A septic systems, and to ultimately inform the feasibility of their wider use in nitrogen pollution mitigation, we conducted a series of focus groups in southeastern Massachusetts where I/A septic system pilots have taken place. This more nuanced understanding of the most relevant considerations to adoption will help resource managers and decision-makers focus on target issues to form effective messaging and outreach, and design behavior change interventions to influence social norms and encourage adoption.

Insights into homeowners’ experiences with and perspectives regarding I/A systems can inform when and where to encourage adoption, how to better communicate what these systems are and the process of adoption to the public, and how to address the concerns and uncertainties homeowners described throughout the adoption process. To understand and compare the types of factors that influence individual decision-making around the adoption of a new technology (I/A systems), we turn to literature on the adoption of agricultural conservation practices (BMPs), household solar (photovoltaic; PV) adoption, and the adoption of electric vehicles (EVs). These technologies are a good reference for our I/A study as they have longer research histories, and the unit of decision-making for adoption is the individual home or farm. Many BMPs, PVs, and EVs also require high upfront costs for a long-term personal investment, much like I/A septic systems. Comparisons to these studies are drawn throughout the results and discussion section. These studies highlight the importance of not only market availability determining technology adoption, but also the influence of individuals’ perceptions of these innovations and other psychological and sociological factors in determining adoption.

## 2 Methods

To identify considerations influencing the adoption of I/A septic systems and to learn from homeowner experiences, we conducted five focus groups from July through September of 2020 (Krueger, 2002; Barbour, 2008; Nyumba et al., 2017). The focus groups lasted 1.6 hours on average and consisted of three parts: the completion of a Q-sort, a brief one-on-one interview, and a semi-structured group discussion. To inform the development of focus group materials, we conducted several informal interviews with I/A pilot implementers and wastewater professionals, observed homeowner meetings, and conducted a literature review of peer-reviewed journal articles and gray literature including news articles related to BMP adoption and behavior change, and analyzed transcripts from Mulvaney et al. (2023) past interviews with decision makers and managers on perceptions of nutrient-reduction technologies including I/A systems. Together, this content was used to create group discussion questions and Q-sort cards. The focus group script and content were pretested multiple times with volunteers and a support team contracted to assist with the focus groups. While we intended to conduct focus groups in person, we modified processes and materials to accommodate a virtual format on the Zoom platform due to the COVID-19 pandemic.

Focus groups started with the application of Q-methodology. Q-methodology is a semiquantitative research method employing inverted factor analysis to reveal a plurality of existing perspectives or opinions of a subject, and how people prioritize those perspectives (Webler et al., 2009; Watts and Stenner, 2012). Participants sorted statements about I/A adoption along a continuum of agreement, called a Q-sort, in response to a guiding question. Typically, these Q-sorts are statistically analyzed to reveal patterns in the way individuals associate opinions related to the prompt (Watts and Stenner, 2012). We used the University of Wisconsin’s Q-tip software for the virtual Q-sorts. The shift to a virtual format impacted the interpretation and usability of the Q-sort data; we chose not to conduct a factor analysis due to participants’ difficulty navigating this tool, software issues, and several incomplete Q-sorts. Absent the factor analysis, these Q-sorts were still useful in corroborating perspectives and opinions revealed during the group discussion based on statements that were most frequently placed in the “least like how I think” and “most like how I think” columns of the response grid. Information on the Q-Sort procedures is included here because the statements served as conversation prompts for both the interviews and the focus groups.

In the second part of the focus group, researchers held brief semi-structured interviews (Patton, 2002; DiCicco-Bloom and Crabtree, 2006; Adams, 2015) with the individual participants. Researchers were paired with a participant and were sent to separate break-out rooms for brief one-on-one interviews. These interviews focused on Q-sort statements participants felt strongly about, found confusing, and other insights from the sorting process (Table 1). For those that had installed I/A systems, this also provided an opportunity to ask about financial aspects of I/A adoption they may not have felt comfortable sharing during the group discussion. The third part of the focus groups entailed an hour-long more traditional semi-structured group discussion, where a set of pre-established questions were posed to the group. The group discussion enabled participants to share their experiences, learn from each other, ask questions, and enabled researchers to follow up on relevant topics brought up in the interviews (Patton, 2002; Nyumba et al., 2017; Gundumogula, 2020). The complete focus group guide, along with focus group instructions sent to participants, Q-sort statements, individual interview questions, and semi-structured discussion questions, the focus group recruitment letter, and the screener for recruiting participants are located on an online repository^6^.

We used purposive sampling to select focus group participants. The geographic focus was on existing, in progress, and proposed I/A septic pilot sites primarily on Cape Cod, but also throughout Massachusetts. We relied on research partners and a professional focus group facility to recruit from lists of homeowners that pilot organizations had contacted to install I/A septic systems, or that had been required to install an I/A system outside of a pilot project. The focus group contractor recruited participants by phone and provided follow-up instructions through email. Participants in focus groups 1-3 were paid for their participation and a focus group contractor recruited the group and ran the Zoom platform, while EPA researchers identified the potential participants for recruitment, developed the focus group guide, and facilitated the focus groups. Participants in focus groups 4 and 5 were not paid and EPA researchers conducted these focus groups fully. For these focus groups, participants were initially contacted by email through EPA research partners, and EPA researchers continued the recruitment process through email. We spoke to adopters and prospective adopters that had:

- installed an I/A system within the last five years to comply with local regulations,
- participated in an I/A pilot in West Falmouth or Tisbury, Massachusetts,
- been contacted to participate in an I/A pilot in Barnstable, Massachusetts in its initial stages of implementation, or
- been recruited but declined to participate in an I/A pilot.

Recruiting from pilots at different stages of adoption and installation enabled us to study homeowners’ experiences with I/A adoption longitudinally. In total, 28 homeowners from 25 households participated in the focus groups: 15 adopters, 11 prospective adopters, and 2 non-adopters. At least seven homeowners were required to install I/As due to local regulations, at least three of which installed systems as part of a group pilot, thus benefiting from a financial subsidy. Though attempts were made to recruit more non-adopters, we were unsuccessful as it was challenging to recruit homeowners to discuss something they chose not to do.

Focus groups were recorded, transcribed, anonymized, and transcripts were imported into NVivo 12 Plus software for coding and analysis. Researchers discussed focus groups and transcripts to identify prevalent emerging themes (Krueger, 2002). We employed intercoder reliability to remove researcher subjectivity and ensure consistent interpretation from coding by having two researchers separately code two focus group transcripts based on an established codebook to 90% similarity. Structural, descriptive, elemental, affective, and some provisional coding were employed following a qualitative coding manual (Saldaña, 2009).

A framework mental model was developed for this data which applied concepts from an integrated model of behavior change as applied to BMP adoption in Reimer et al. (2012). We use this general integration to illustrate the importance of both background factors and adopter perceptions in technology adoption.

## 3 Results and discussion

**FIGURE 2:**
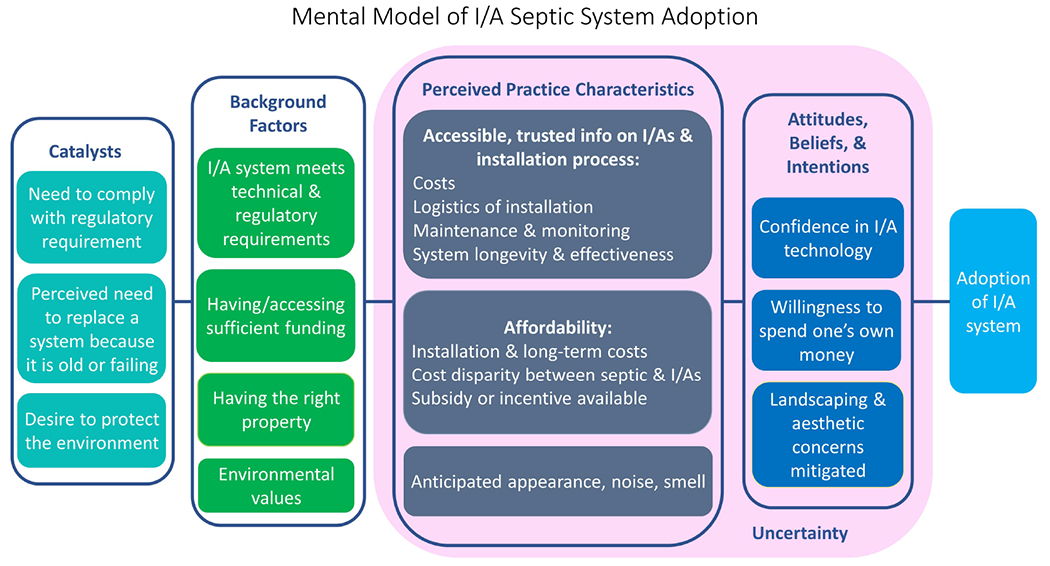
Figure illustrating a mental model of homeowner decision making around I/A system adoption. The types of factors relevant to homeowners’ decision-making are categorized as catalysts; background factors; perceived practice characteristics; and attitudes, beliefs, and intentions. This structure applies concepts from Reimer et al.’s (2012) model of BMP adoption which integrated elements of the Diffusion of Innovations and Reasoned Action Approach

The factors and conditions that influence homeowners’ decisions to install I/A septic systems are illustrated in Figure 2, a conceptual, collective mental model of I/A adoption. We modified the model from Reimer et al. (2012) as a framework for describing and organizing the psychological and contextual elements that interact to shape decision-making around the adoption of I/A systems. This mental model was used to frame focus group results and accompanying discussion. It is a representation of how adopters and prospective adopters make the decision to adopt an I/A system. While we intended to analyze homeowners’ perspectives longitudinally, analysis revealed no notable differences between the adopters and prospective adopters, leading to analysis of all participants together. Mental models are internal representations of external reality that individuals use to interact with the world around them, and explain how individuals reason, make decisions, and, to a certain extent, how they behave (Jones et al., 2011). The factors that shape perceptions and decisions are plentiful and complex and are ultimately difficult to adequately capture in one useful model. This model, therefore, is not a predictive model and is not intended to comprehensively identify and characterize all possible factors that influence decision-making. Rather, it illustrates the different types of major considerations that homeowners perceive to be important when deciding to adopt an I/A septic system. Because adopters and prospective adopters had similar perspectives, only one mental model was created.

This model describes the I/A adoption homeowner decision making process from identified catalysts; background factors, perceived practice characteristics, and attitudes, intentions, and behaviors, described in more detail as follows:

- Catalysts are factors that drive homeowners to consider adopting I/A systems in the first place. These include the perceived need to replace a septic system, the regulatory requirement to install an I/A system, and a desire to protect the environment. These factors often worked synergistically to catalyze interest.
- Background factors describe an array of personal characteristics, property characteristics, underlying values and personality traits, and the larger social, political, and economic context within which I/A adoption is taking place. These pre-existing conditions and considerations are not influenced by the process of adoption. Instead, they influence behavior by determining choices and alternatives to adopting an innovation. Background factors directly influence perceived practice characteristics, and in turn influence behavior by indirectly influencing attitudes, beliefs, and intentions (Fishbein and Ajzen, 2010; Reimer et al., 2012). The literature on agricultural BMPs finds the context within which a farm operates (a background factor), influenced by variables such as commodity prices, local climate, the availability of government funds for adoption, and policies, influences farmers’ willingness to adopt BMPs (Reimer et al., 2012; Pannell et al., 2014; Liu et al., 2018).
- Perceived practice characteristics describe how individuals perceive an innovation or practice (Reimer et al., 2012). The actual characteristics of an innovation are less influential than how an individual perceives these characteristics, as individual perceptions influence behavior (Rogers, 2003; Reimer et al., 2012). If these considerations are not perceived to be problematic, these perceived characteristics facilitate adoption. If they cannot be addressed, they become constraints. The perceived practice characteristics are influenced by background factors, and in turn influence attitudes, beliefs, and intentions. The perceived practice characteristics identified in this study are consistent with those identified in the DOI (Rogers, 2003), and as used in a similar study on wastewater technology adoption in Florida (Warner et al., 2022). These include 1) the relative advantage of an innovation over alternatives; 2) compatibility, perceived consistency with an individual’s existing values and needs; 3) complexity, the ability to be understood and used; and 4) observability, the degree of visibility to others (Rogers, 2003). The DOI and Reimer et al. (2012) also include trialability, or the degree to which an individual can experiment with an innovation, as an important characteristic, but is not possible to consider trialability in the case of I/A systems because they are expensive and require significant modifications to properties. Trialability has therefore been excluded from this analysis as it was for the Warner et al. study on factors driving septic to sewer conversions (2022).
- Attitudes, beliefs, and intentions are socio-cognitive elements which interact to influence behavior (Fishbein and Ajzen, 2010). Attitudes describe homeowners’ enduring positive and negative predispositions (Kollmuss and Agyeman, 2002) about these systems. Beliefs describe a knowledge base or personal truths upon which attitudes are formed (Cary et al., 2001), and intent describes a homeowner’s intention to adopt. This study did not define nor measure specific attitudes, beliefs, and intentions surrounding the adoption of I/A systems, so we have grouped these elements of behavior into a single broad category. We identify three major factors influencing this category of ‘Beliefs, Attitudes, and Intentions’: 1) confidence in I/A system technology; 2) homeowners’ willingness to spend their own money; and 3) the belief that concerns related to landscaping, aesthetics, and the noise and smell emanating from a system can be addressed. These are the outcome of the catalysts, background factors, and perceived practice characteristics associated with adoption.
- Uncertainty describes the risk and uncertainty homeowners perceived related to I/A systems and the process of installation. While most participants did not necessarily describe the installation of I/A systems as risky, some degree of uncertainty could be attributed to nearly every factor that influenced homeowners’ decision-making. For this reason, in this model uncertainty encompasses both perceived practice characteristics and beliefs, attitudes, and intentions, despite being considered only a perceived practice characteristic in the literature (Cary et al., 2001; Reimer et al, 2012). Uncertainty will not be explored as a standalone section as it is discussed throughout each component of the Perceived Practice Characteristics and Attitudes, Beliefs, and Intention sections below. Due to the “newness” of an innovation, uncertainty is an inherent part of diffusion (Rogers, 2003). Studies on the adoption of agricultural BMPs, climate-friendly practices (Wreford et al., 2017), the adoption of energy-efficient technologies (Wilson and Dowlatabadi, 2007; Wood et al., 2016), and solar energy point to uncertainty or risk as a relevant factor in adopters’ decision-making. Uncertainty about land tenure, the installation of a BMP given site heterogeneity, crop yields stemming from BMP adoption, and the skills required to adopt a BMP are just a few examples of how uncertainty can discourage the adoption of BMPs (Cary et al., 2001; Greiner and Gregg, 2011; Liu et al., 2018). As to solar energy, uncertainty related to government policies, pricing and funding, the lifetime of solar panels, among other factors, are several ways in which uncertainty can deter adoption (Shih and Chou, 2011; Mundaca and Samahita, 2020). As a relatively new innovation at the household level with high initial costs, there are critical parallels for I/A adoption to all these uncertainty concerns making reducing uncertainty throughout the process an important component of wider implementation.

### 3.1 Catalysts to the decision-making process

#### 3.1.1 Perceived need to replace a system & regulatory requirement

The need to replace an existing onsite wastewater treatment system to comply with regulatory requirements often dictated by local environmental regulations, and the perceived need to replace a system because it was old or failing, were two of the primary catalyzing factors prompting homeowners to consider I/A adoption. While some homeowners installed I/A systems voluntarily, as one homeowner put it, “there are very few people out there that are going to replace their septic system just for the sake of replacing their septic system. They are replacing the septic system because it has either failed or they’re rebuilding or renovating their house in such a way that they need to reconstruct their septic system.” Those mandated to install I/A systems were required to because they were located or building in a nitrogen-sensitive area and needed to install the system to comply with zoning regulations, and/or had purchased a house with an existing failed system or cesspool. It was difficult to tease out how many of these conditions applied to homeowners that were required to install: for some, a number of these conditions applied but they were not asked to identify all conditions for required upgrades they may have met.

Several of the participants had to install an I/A system as a condition of renovation. “We did a demolition and reconstruction project in [town] and none of the houses that we demolished had a compliant septic system. Nobody was going to do Title 5 and sell those houses. I was going to have to put in a new septic system anyway,” mentioned one homeowner. Another participant who had recently purchased a house from 1850 added, “I’m the first person to buy [my house] in 125, 150 years so it [on-site treatment system] was actually just a pit which they don’t even allow anymore. I’m redoing that and hence we needed a new septic.” Yet another homeowner installed an I/A to realize the full potential of their house, which had five bedrooms but was only permitted for three. “We didn’t have to do [it], but we had to do it if we wanted to be able to accommodate our entire family and friends … and we can rent it out and have friends over and do all these things we otherwise wouldn’t have been able to do,” they shared. Only two homeowners installed an I/A system to permit an additional bedroom, which was surprising as we had anticipated this to be a more popular reason motivating adoption.

Others installed I/A systems to comply with local environmental regulations surrounding nitrogen-sensitive areas and waterbodies. One of these homeowners’ property was located “within the 100-foot buffer zone” in a nitrogen-sensitive area. “Motivation for the I/A system was to purchase my property. There is no option, I had to have an I/A system … We don’t have a choice if you live on the water,” they stated. “Our backyard abuts that marshland, wetland. I didn’t have a choice in the matter. The board of health mandated that I get an I/A system,” shared another.

Those who voluntarily installed an I/A system described wanting to replace an old septic system or cesspool. “We wanted to get rid of our old cesspools even though they were working fine. I didn’t like that,” explained one homeowner. Another shared, “I told the [pilot organization] ‘when you get over to my side of the bay I want to be involved’ because our existing system was [from] 1969 and ‘un-permittable’ by today’s standards and we live right on the edge of the salt marsh.” This was true among the prospective adopters, too. “We have a working cesspool from 1969 and it’s in perfect condition, which we were pleased with, but you know if we could upgrade it to something that is state-of-the-art, that would be great for the pond which would be wonderful,” stated one participant.

#### 3.1.2 Desire to protect the environment

It became apparent throughout the focus groups that synergy existed between the perceived need to replace a septic system and a concern for water quality as drivers of installation. For many participants, the opportunity to do something they perceived would protect the environment, that might specifically improve local water quality, was an important catalyst to adoption. “My system was failing. If I was going to replace it, I was going to upgrade and accomplish the goal of improving the water quality … There was no question in my mind that I wanted to do something that would contribute to … West Falmouth Harbor,” stated one homeowner. Required to install an I/A system for reasons of environmental compliance, another homeowner shared that when they had learned about water quality issues locally, “Then [installing the system] became an issue of the water quality is bad and we need to help do something about that.” Another prospective installer expressed a desire to upgrade out of concern for the deteriorating water quality in their neighborhood, stating, “I don’t want to be a contributing problem to the problem. You feel like you should go downtown when you have to pee so you don’t have to pee in your own neighborhood!” This concern for local water quality was especially poignant for adopters who participated in pilots led by non-profit environmental organizations. Some perceived the installation of an I/A system to be the only way to mitigate problematic nitrogen inputs to the coastal zone.

### 3.2 Background factors

#### 3.2.1 I/A system meets technical and regulatory requirements

For an I/A system to be considered a candidate for onsite wastewater treatment in Massachusetts, it must meet the technical and regulatory requirements set forth by the state and the town where the system is being installed. Currently, I/A systems must meet a regulatory discharge standard of 19 mg/L of nitrogen to be considered for use in Massachusetts compared to the 26 mg/L of nitrogen assumed to be discharged from traditional septic systems (Title 5 of the State Environmental Code, 310 Code Mass. Regs. §§ 15.000). Other technical and regulatory requirements related to the construction and replacement of onsite systems with I/A systems are detailed in sections 15.280-15.288 of 310 CMR 15.000: Title 5 of the State Environmental Code.

#### 3.2.2 Having access to sufficient funding

Having access to sufficient funding is a prerequisite for the installation of an I/A system. While most focus group participants received a financial incentive to install an I/A system, all were required to contribute to their installation to a certain degree. This is corroborated by a homeowner who paid more than half of their installation, sharing “With the engineering, the permitting, and then the installation, I think the total was $26,000 and the grant covered $10,000 of that. We felt we could afford that but, I can’t see a young couple with kids [installing an I/A].” Another homeowner who had received $10,000 towards an installation that cost them an estimated $40,000, explained that they had been concerned about nitrogen and pollution long before installing their system, but had kept their functioning cesspool because they “didn’t have enough money at that time” to replace it with an I/A. Purchasing, installing, maintaining, and monitoring an I/A septic system is expensive and often requires access to funding even after a financial incentive is provided.

#### 3.2.3 Having the right property

Whether a homeowner has the right property for a particular I/A system is a function of site constraints. Lot size and layout, including the locations of obstacles like boulders and trees, overlap with wetland buffers, and depth to groundwater can determine whether a specific system is appropriate and where it might be placed on the property. Several homeowners were required to adapt the installation of their system to accommodate spatial limitations. Upon describing why their I/A system had to be moved, one homeowner described, “When they dug up my house, there were a couple of Native American possible burials so we actually had to move the leach field a little bit over to accommodate that so they wouldn’t get affected.” Others had site constraints that required their systems to be placed under their driveway.

#### 3.2.4 Environmental values

In this study, ‘environmental values’ is used to convey that many participants possessed strong and deep-rooted sentiments related to protecting the environment. Values (a general worldview) are more durable aggregations of beliefs and attitudes about what is important to a person, providing motivation and guidance by which people behave and make choices (Cary et al., 2001; Steg, 2016). We interpreted the desire to protect the environment and a concern for local water quality as aspects of environmental values. We also interpreted feelings of responsibility towards protecting the environment, and the fact that many of the homeowners that participated in pilots trusted and/or were supporting members of environmentally oriented non-profits, to be indicative of environmental values. Some participants spoke more often about environmental values and considerations than others, but nearly all participants exhibited some element of pre-existing environmental values. For instance, one homeowner talked about installing solar panels years earlier, another referenced work they had done with an environmental organization prior to an I/A pilot’s existence, and another talked about their role in helping connect an environmental non-profit with other clients.

Environmental values were most obvious among homeowners that discussed a concern for water quality as their single most important factor driving installation. This concern was rooted in feelings of responsibility for protecting water bodies that participants lived on or valued for recreational, aesthetic, or cultural reasons. “I’ve seen the degradation of the cove and harbor markedly over that period of time; it’s effectively dead … I did it out of a moral concern that I enjoy the water that I live and play on, and felt I had the resources economically and that it was the right thing to do,” stated a homeowner who decided to install an I/A system after declining to participate in a pilot. “My wife and I decided that we owed that to the bay for the enjoyment that it has afforded us and our families historically and prospectively,” they added. Another homeowner shared, “My motivation is that I’m right on the edge of a small pond. With the beach and the bay right beyond that I’ve been concerned for a long time about the problem of nitrogen and pollution generally.” Environmental values were also evident among homeowners that were mandated to install I/A systems independently of a pilot. “I really am worried about the water quality. The planet but also in our backyards,” shared one of these homeowners, and “We want them to work. We are doing this because we care about doing our little bit for the environment and if everyone does their little bit then we’ve all done a big bit,” shared another. We had anticipated environmental values to be more prevalent among homeowners that worked with environmentally oriented pilot organizations to install their systems and found that environmental values were an important factor across nearly all homeowners.

The role of environmental values in influencing behavior and adoption is complex, and consensus around this topic is mixed (Dietz et al., 2005; Steg and De Groot, 2012; Schelly, 2014). In an analysis of drivers of and constraints to the adoption of sustainable practices, researchers found that pro-environmental values are overpowered by more influential incentives or disincentives to adopt. They find that these attitudes are strongest when there are no strong external incentives or disincentives to adopt (Cary et al., 2001). An examination of the relative importance of environmental motivations driving the adoption and diffusion of solar panels in Wisconsin found that not only are environmental values insufficient in driving adoption alone, they need not always be present to motivate adoption (Schelly, 2014). However, in a study examining factors driving the adoption of PV in Sweden, researchers found that “higher environmentalism increases the odds of having a higher likelihood of adopting” (Mundaca and Samahita, 2020). Given this lack of consensus, it is difficult to generalize around the role of environmental values in influencing adoption. While we found that environmental values appear to significantly influence homeowners’ decision to adopt I/As, and work synergistically with other factors such as financial incentives to motivate adoption, it is uncertain whether this would be the case in different localities. It is possible that because homeowners from three out of the five focus groups participated in pilots that were implemented by environmentally oriented non-profits, the homeowners they recruited already possessed strong environmental values, which could explain the importance of environmental values in driving I/A adoption in this study. The importance of environmental values in driving adoption could be influenced by background factors such as the social or political context within which adoption is taking place, or how the benefits of adoption are presented. For example, as highlighted in the Wisconsin PV adoption study, framing adoption as pro-environmental could limit adoption in areas where environmentalism is negatively associated with certain political philosophies or affiliations (Schelly, 2014). It is also important to note that measuring environmental values was not an objective of this study.

### 3.3 Perceived practice characteristics

Perceptions of I/As and the installation process are an important component in the spread of adoption of the systems, as highlighted through the basic elements of relative advantage, complexity, compatibility, and observability in influencing the perceived acceptability of I/As (Diffusion of Innovations: Rogers, 2003; Reimer et al., 2012). Compatibility, whether homeowners perceived I/A systems to align with their needs and values, and observability, whether the outcomes of adopting an I/A system were apparent to homeowners, were the most significant influences on acceptability. Similarly, Warner et al. (2022) also found compatibility and observability to be significant predictors of support for septic to sewer conversion programs in Florida. In our focus groups there was less discussion of the I/A systems as a relative advantage over keeping their traditional septic system, but that is likely due to the large participation of homeowners who were required to install the systems or who needed to replace their existing system anyway. The focus groups also consistently reflected the interconnected nature of the characteristics of an innovation. For example, the complexity of the financing, installation. maintenance, and monitoring processes greatly influenced the perceived observability of the systems which then influenced the perceived compatibility. Due to this interconnectedness, we discuss the perceived characteristics together within three general themes:1) access to trusted information on the entire I/A adoption process, 2) perceived affordability, and 3) perceived aesthetic impacts (smell, sound, and visual disruptions).

#### 3.3.1 Accessible, trusted Info on I/As & the installation process

The perception that it was difficult to find comprehensible, locally relevant information on I/A systems was common among focus group participants, affecting the perceived observability of the systems. However, many participants did not go to great lengths to conduct their own research. Not having conducted research on I/A systems was a common thread in most of the focus groups among adopters and prospective adopters alike. Prior to installation, most participants noted they had not even been familiar with I/A systems. Most relied on their contractors or the pilot organization to be informed about these systems. One prospective installer with little prior knowledge of how septic systems work admitted, “No, I didn’t do any research on this at all. As long as the toilet was flushing, the water was running, everything was fine, everything was good,” and later added, “Someone came out and explained it to us, but, you know, it’s a whole new concept.” Although a handful of participants had conducted their own research and appeared to be relatively well-informed, this was the exception. Still, participants shared their insights on a perceived lack of accessible information related to these systems and their installation.

Homeowners were asked in the group discussion whether they had difficulty finding information on I/A systems. Some signaled a dearth of general information on these systems. “I would have been very interested to know more about it before getting into it if the information was there,” suggested a homeowner required to upgrade for new construction. Others were more specific, discussing a lack of information about system costs, longevity, monitoring and maintenance responsibilities, and a lack of regionally specific information on these systems. A homeowner familiar with the installation process given their related occupation explained some of the sentiments around information access:

“I think there is confusion [about] the different systems that are out there. I think for the layman walking into it, trying to navigate the different systems, their pluses, their minuses whether it be operational or initial capital cost and then, the third leg being monitoring is a lot for I would say 90% of the consumer homeowners out there to take on.”

##### 3.3.1.1 Information on system costs

A lack of information on the overall cost and different types of costs associated with installing an I/A system was revealed throughout the focus groups. Several adopters and prospective adopters noted that prior to installation, they were unaware of what it would cost to purchase and install a system. One homeowner even asked researchers in a focus group, “how much does it cost to install?” Some had anticipated that the process would be costly, and some had not. “I knew it was going to cost me a fortune and it did,” reflected one homeowner. Another recounted their reaction when finding out about these costs, stating “When I first got the initial quote, I flipped out and said ‘you gotta be kidding.’” The homeowner was able to work with the contractor to make the cost more acceptable. One homeowner lamented, “We would not have laid out what ultimately ended up being six figures over a period of a few months to go from a 3-bedroom septic to a 6-bedroom septic.”

Beyond initial installation costs, there was also a lack of information on long-term and continuing costs. A homeowner who installed a system independently and whose biggest barrier was cost and continuing costs stated, “No one could give me an idea of what it was going to cost to run this system month to month.” Recent adopters that had participated in a pilot echoed this uncertainty regarding long-term and maintenance costs, which is likely attributable to the pilot organizations being responsible for these costs for the first several years after installation.

##### 3.3.1.2 Information on logistics of installation

Upon being asked to describe their experiences before, during, and after I/A system installation, it became clear that most homeowners lacked an understanding of what installation would entail. This was especially apparent among the prospective installer group. Upon being asked whether there was anything they felt did not know about the process of installation, one prospective installer responded, “I probably don’t understand anything. I just know that it’s a big, big hole … a big excavation … I would like to know where it would be in my backyard.” Others had questions about what these systems would look like on their property, how system placement would be determined, and the duration of installation. When it came to system siting, participants described having to consider smell, landscaping characteristics they wanted to preserve, and driveway placement. For many homeowners, installation logistics and system appearance remained unknown until installation was complete.

##### 3.3.1.3 Information on maintenance and monitoring

Homeowners expressed concern and uncertainty when discussing system maintenance and monitoring, which could be attributed to many not having been responsible for maintenance and monitoring yet, which was covered by pilot organizations for the first several years. “Getting it monitored periodically; wear and tear on the maintenance was my concern,” described one homeowner. “I’m a little concerned about future costs and our responsibility as the homeowner to schedule check-ups and maintenance and all that,” admitted another. One homeowner was also worried about their ability to find someone with the knowledge to maintain their system given its novelty. Though uncertainty around monitoring and maintenance characterized most perspectives, there were exceptions. A homeowner knowledgeable about onsite wastewater treatment systems (OWTS) felt strongly that monitoring and maintenance would be a burden given their situation. They explained that as a seasonal homeowner, it would be a challenge for them to keep their system compliant with the low flows and episodic flows associated with seasonal homeownership. “You need to have waste in order to have bugs, in order to break down the waste and consume nitrogen,” they described. Homeowners considered the longevity of I/A systems when deciding whether to install, an aspect of the relative advantage of adopting I/A systems over Title 5 systems, although discussions around longevity and effectiveness centered around a lack of information on these subjects. A homeowner that perceived I/A system complexity and longevity to be “risky” explained: “I struggle to find data on long-term studies on these types of systems. We know how long cesspools last or Title 5 lasts because there are so many data points and they’ve been building them for so long … Do we know if they [I/A systems] continue to work in the way we want them to continue to work in 8, 10, 25, 30 years down the line?”

Risk perceptions around system longevity could be partially attributed to a lack of long-term maintenance, monitoring, and performance data. This data does not exist for some of the more novel systems being installed, which have not been installed in large enough numbers to produce scientifically backed estimates on longevity (Cape Cod Commission, 2015; Great Lakes Environmental Center, Inc, 2021).

##### 3.3.1.4 The role of pilot organizations as purveyors of trustworthy information

Trust in the organizations coordinating and implementing I/A septic system pilots was an influential factor in determining whether homeowners perceived they had access to accessible and trustworthy information on I/A septic systems, influencing the perceived observability of the systems. Whether homeowners trusted a pilot organization was not explicitly solicited as part of the focus groups but was an emerging theme. This trust manifested itself in two ways: 1) trust in the pilot organization to handle installation-related logistics and, 2) trust in the pilot organization as the disseminator of information.

Trusting the pilot organization to handle installation logistics reduced homeowners’ perceived complexity, by enabling them to forego the burden of researching and learning new information they would have needed to navigate had they installed an I/A system outside of a pilot. In describing the role of the pilot organization, one homeowner mentioned “They were incredibly helpful. I basically didn’t have to do anything. They managed the whole process for me.” Others relied on pilot organizations as purveyors of information related to these systems, often citing informational meetings or sessions hosted by the pilot organization as their only source of information. Trust in the information provided by pilot organizations became evident when participants suggested having a “good foundation” or feeling “well-informed” from having attended meetings hosted by the pilot organization or choosing a system because a pilot organization “was excited about [it].” Some homeowners placed significant trust in the information coming from pilot organizations, and one explained:

“I have no reason to believe that [pilot organization] would tell us something that wasn’t true. And the system they have that they were talking about seems very simple. Don’t know exactly what it’s going to cost, have not heard anything about that, but it didn’t sound like it was risky as far as the technology. So, there’s not enough risk to stop me from doing this…”

As disseminators of perceived trustworthy information, non-profit organizations can support broader-scale adoption of I/A systems. In a report identifying knowledge gaps for Hawai’i’s cesspool conversion plan, Mezzacapo (2020) suggests that partnering with local organizations that have similar state or watershed-level objectives, such as managing land-based pollution and increasing awareness, may be advantageous for homeowner outreach. This research supports that finding and highlights the need for collaboration across institutions for broader implementation.

##### 3.3.1.5 A need for better outreach and education around I/A systems

Several participants stressed the need for more education and outreach on I/A systems, a need echoed in a survey of OWTS programs by Hawai’i Sea Grant (Mezzacapo, 2019). One of the participants mandated to install an I/A system indicated that had they previously known about these systems, they would have installed one anyway. “It would be better if there could be more education available for people,” they stated. “If you tell me I can buy a car that’s going to pollute a whole lot, and a car that is not going to pollute a whole lot, and the non-polluting one is going to cost more- I’m going to go for that car that does not pollute more. I’m going to pay the extra money because I care.” Another homeowner recounted an interaction at a town meeting for a building permit application. They had witnessed a homeowner being granted a permit contingent on installing a functional septic system, and expressed frustration when an administrator told them it was not required to notify homeowners about the option of I/A systems at these meetings, stating:

“I’m afraid there is too little information. People don’t even know about it [I/A systems]. … I think there needs to be a lot more publicity and a lot more education and a lot more participation by the local authorities. They don’t even suggest it when people need it and are located right down here on the water!”

To document specific informational needs, we asked participants, “Is there anything you know now about I/A systems, or about the process of installation, that you wish you knew prior to installation?” and asked participants to briefly describe their experience prior to, during, and after installation. We’ve summarized responses in Table 2, which classifies homeowners’ informational needs by type. For some homeowners, these needs did not arise until installation was underway or already complete, though ideally this information would be provided prior to agreeing to install an I/A system and included in communications directed at potential I/A adopters.

#### 3.3.2 Perceived affordability

We identified three factors that ultimately influenced homeowner perceptions of I/A systems’ affordability: 1) the total cost of adoption including landscaping and long-term operation and monitoring costs, 2) the cost disparity between traditional septic systems and I/A systems, and 3) whether a subsidy or financial incentive was made available. The cost associated with buying and installing an I/A system was the most prominent consideration inhibiting adoption. This is consistent with the literature on the adoption of other capital-intensive technologies with high up-front costs such as solar or electric vehicles (Margolis and Zuboy, 2006; Adhikari et al., 2020; Krishna, 2021). By mitigating installation costs, the provision of financial incentives was among the most prominent motivators of adoption beyond the perceived need to replace an old or failing existing system. Throughout the focus groups, cost was discussed in general terms, as participants had difficulty breaking down costs and attributing them to different components of installation. This was because some homeowners took advantage of excavation to make non-required modifications to their property and because the financial aspects of installation had been more heavily managed by the pilot organizations. Despite difficulties breaking down exact costs, participants discussed the types of costs they confronted.

Although costing data for the full installation and monitoring is scarce, I/A systems themselves cost significantly more than traditional septic systems (Cape Cod Commission, 2015). The cost burden of these systems was a common discussion point in every focus group. All participants, without exception, perceived the adoption of an I/A system to be expensive, and referred to this cost as a burden to one extent or another. “I’m sure you didn’t want to pay 30k for something that goes underground you don’t see, you know? It would be a hard pill to swallow, I think, for a lot of people,” shared one respondent who felt very fortunate most of their costs were covered. “Some people just can’t afford it,” stated a prospective installer, a sentiment that many participants shared. Others referred to the cost of I/A systems and their installation as “extremely high,” “an issue,” “an expensive situation,” and “a big obstacle for a lot of people.” “If you can get those costs down another 25-35% I think they would be much more appealing,” suggested a homeowner.

##### 3.3.2.1 Total cost of adoption

Estimating the total cost of adoption is difficult because different costs are extremely variable depending on site and individual characteristics, landscaping, and additional work pursued during excavation. Homeowners had difficulty parsing the individual expenses associated with installation in some cases because installation was rolled in with other site work on a property. The average figure reported was between $30,000-$40,000 USD prior to the financial incentives. Figures ranged from $22,000 to over $100,000, the latter was reported by two respondents who made additional unrelated adjustments to their property or who had to purchase additional land to comply with local environmental regulations.

Landscaping costs were difficult to pin down. These costs are influenced by variables like the size of a property and the degree of landscaping being pursued, and how one defines landscaping. Definitions of landscaping ranged from replacing lawn, to building walls and removing trees, to excavation costs. Several participants discussed a range of costs for landscaping. One homeowner shared, “We did a lot of extra work like burying utilities and we had a generator that we pump out to the leaching field. It was between $35-$40,000 when we were done. I guess with landscaping and engineering and plumbing we actually spent $45,000.” After having spent an “enormous amount of money on building a new wall in order to regrade a whole section of property,” another homeowner surmised that their landscaping bill was higher than what most people pay for their traditional septic system. Yet another cited landscaping as expensive because they had to rip up a lot of the landscaping they had previously done, though another stated they didn’t have to spend any money on returning their landscaping to its original state as promised by the pilot organization.

Like landscaping costs, long-term costs associated with operation, maintenance, and monitoring also factored into homeowners’ perceptions of affordability. Discussion was centered on general uncertainty of future long-term costs. “I’m a little concerned about future costs,” shared one homeowner. Homeowners were asked to describe the long-term cost of monitoring and maintenance, and whether these costs were what they had anticipated. Most had not yet confronted these costs, stating “I’ve had none,” “ours [system] is new,” and “we are not paying anything for monitoring, and we haven’t had the maintenance yet.” A minority of homeowners were able to provide figures for their long-term costs. These ranged from $500 to $3,000 a year. While some did not find this to be a financial burden, others did see this continuing cost as a major risk in I/A adoption. “My biggest risk was cost and continuing cost … I don’t make that much money so adding another $1,200 a year just to maintain my system which already costs a fortune [is a lot],” shared one homeowner. Another mentioned they spent $500 per year on maintenance and monitoring, “It is consistent with what it was costing us to pump out the failing septic system the last couple of years. It’s not been a burden.” A third explained that they had included long-term costs into their system contract, which amounted to $3,000 per year. “I just wanted it dealt with in one big hard band-aid to want to take off … So, I added it to the cost of everything.”

##### 3.3.2.2 Cost disparity

The cost disparity between traditional septic systems and I/A systems also influenced the perceived relative advantage of affordability. One non-installer stated, “People are going to have different things to motivate them. For some people it’s going to be purely coming down to the money. Does it cost more? If the answer is yes, they are not going to want to do to it.” Another noted, “The cost of an I/A system versus a conventional gravity system: there is such a disparity there. The gap needs to be closed. I can’t imagine anyone without getting incentives or without being forced to do that they would actually go ahead and do it.” One homeowner mentioned rhetorically, “If somebody has a failed septic system [and] if the cost can be the same, then why wouldn’t you do the upgrade?” These highlight the value of financial incentives as a powerful tool to make I/A and traditional systems cost-competitive.

##### 3.3.2.3 Financial incentives

Financial incentives made upgrades to I/A systems more economically feasible for many homeowners and emerged as one of the strongest motivators of adoption. This is consistent with the role of incentives or subsidies described in the adoption literature on PV adoption and agricultural BMPs (Sierzchula et al., 2014; Liu et al., 2018; Mundaca and Samahita, 2020). One respondent explained, “Money motivates behavior. Money affects behavior. We all know that; it’s a fact.” For some homeowners, the financial incentive presented an opportunity to install a system they otherwise could not afford. One homeowner described being previously unable to replace a functioning cesspool with an I/A system due to unaffordability prior to enrolling in a pilot, “This time I had to do something. This is an opportunity to take care of it properly on my property.” Although financial incentives emerged to be the strongest and most cited motivators of adoption, it is important to recognize they worked in tandem with catalysts to encourage adoption. “If you are going to replace failed systems, you’re going to be spending a lot of money anyway. So, the fact that [pilot organization] kicked in $10,000 made it a really easy decision,” stated one homeowner. Another striving to replace a cesspool echoed this notion, suggesting that the financial incentive being offered encouraged them to participate in a pilot. Yet another mentioned that the financial incentive allowed their family to upgrade to a system that would allow them to continue to occupy their home, rather than “buy another place on the island.”

#### 3.3.3 Anticipated appearance, noise, and smell

Aesthetic and landscaping concerns factored into homeowners’ decision-making, though for most homeowners, they did not prove to be a dealbreaker when it came to adoption. Aesthetic considerations encompassed the anticipated appearance of a system: how the I/A systems physically look and how the systems might change the current appearance of a property. The focus group participants expressed general uncertainty about the anticipated appearance of these systems prior to installation. Others were more explicit when discussing their expectations. One homeowner feared the I/A system would be “a bizarre thing that was in the yard.” They explained, “Aesthetics are important to us and the big concern to us was what is it going to look like? Is it going to be like … these weird pipes sticking out of the ground, or are we going to be able to notice it?” Another homeowner recounted, “We didn’t want that PVC U pipe coming out of the ground,” though these were not part of the system installed.

Several homeowners were surprised by the resulting system and landscaping aesthetics associated with I/A system adoption. One installer described their wife’s disappointment when three manhole covers were placed right in front of their driveway. “It’s not a big deal for me, but my wife was completely surprised. This is her dream house and now she’s got these manholes. If I would have known that it would have softened the blow for her perhaps,” they explained. Another participant that had undergone complex landscaping shared, “It changed the state of our property a little. We had to take down a couple of old trees which was pretty hard to do but there was no other way of fitting the system in without taking those trees out….” Yet another described, “We have a sewer cover in our back lawn, and it wasn’t very pretty.”

Several homeowners perceived that noise and smells associated with these systems could be problematic, but these perceptions had little influence on I/A system adoption. This was somewhat surprising given that wastewater managers and industry representatives who participated in informal interviews as part of Mulvaney et al. (2023) research had previously identified concerns related to noise and smell to be relevant to homeowners. Of the twenty-eight homeowners we spoke with, only one cited noise as a major concern, and several others acknowledged it was a reality but did not describe noise as an inconvenience. Among the few that discussed these concerns, none had anticipated these concerns prior to adoption but instead became aware of the potential for noise and smell during the installation process through discussions with their engineers or installation contractors. One homeowner stated the noise from their system was “not well publicized.” “The noise from the exterior pump was not something that I read about or heard anybody complain about,” recalled this homeowner, who became aware of the potential for noise only after their contractor pointed it out. As far as smell, few homeowners described this aspect to be a nuisance. “The only issue that we’ve had with [the I/A system] was that during some hot summer days some of the exhausts was a little smelly,” recalled one homeowner.

### 3.4 Attitudes, beliefs, and intentions

Attitudes, beliefs, and intention interact to influence the individuals’ willingness to adopt I/A systems (Fishbein and Ajzen, 2010). For this study, we have grouped these into one category and identified three major contributing factors: confidence in the I/A technology, willingness to spend money, and the belief that negative aesthetic impacts can be mitigated.

#### 3.4.1 Confidence in I/A technology

Although there was a general lack of familiarity with I/A systems, most homeowners expressed confidence in I/A systems’ technical abilities, a confidence shaped in part by the pilot organizations that communicated on these technologies. This confidence positively influenced the adoption of these systems. While we had anticipated that homeowners would approach these systems with more skepticism given their relative novelty, this was not the case. “At the end of the day it’s a system that works. We’re told that it’s a system that is doing what it should do,” offered one participant. Another stated, “I guess I believe in the system. I mean, I have to believe in it. The engineering is there.” Yet another exclaimed, “I didn’t see any big risk. Tried and true systems!” Prospective adopters shared this confidence even with their lack of hands-on familiarity with the installation process. Despite “being in the weeds on how these systems work,” a prospective installer offered, “I think we would agree that there’s research out there that suggests these would help the environment.” Another said, “I think the issue will be the financing. But the system makes sense, everything I’ve heard about it- my son did a lot of education with me, and they explained quite a bit to me, so I understood it a little better.” Homeowners that were less optimistic about I/A technology cited concerns related to system complexity, durability, and longevity, that it’s “not a proven technology.” Only two adopters expressed significant concerns regarding I/A technology. “They couldn’t tell me what would happen if the system failed,” one stated. “Given that it was a true, true, true pilot, if it didn’t work, I would have to replace it and that would have been enormously disruptive,” explained another on why they chose not to participate in an I/A system pilot.

#### 3.4.2 Willingness to spend one’s own money

A willingness to spend one’s own money was an important consideration influencing adoption among homeowners, even after being eligible to receive a financial incentive. This willingness is influenced by background factors such as the ability to access funding, timing, competing monetary needs, and a myriad of other personal contexts and situations that exist outside the project. Emotions and opportunity costs, foregoing other goods or services that could be spent with one’s money, also determine one’s willingness to spend money (Carter, 2014). Despite being eligible for a $10,000 incentive, one of the homeowners we spoke to choose not to participate in an I/A pilot for this reason, explaining, “At the moment, I wasn’t ready to spend the money,” as one of two reasons they chose not to install an I/A system.

#### 3.4.3 Aesthetic, noise, and smell concerns mitigated

Finding ways to mitigate and adapt to concerns related to aesthetics, noise, and smell, shaped how homeowners viewed and felt towards these systems overall. For the few that factored these concerns highly, the ability to mitigate these concerns was central to their decision to adopt. Homeowners that were initially dissatisfied with their systems’ appearance found creative solutions to address these concerns. Several had worked with their contractors and engineers to site systems in a manner that preserved important aspects of their property. For example, one homeowner was able to change the placement of their system to salvage old oak trees that were important to their family. Others found ways to adapt to aspects of their systems they did not find appealing. These included decorating manhole covers and building small structures to conceal pumps and pipes. After matching their manhole covers to other aspects of the yard, one homeowner mentioned the system was “a feature not a bug anymore.” Another homeowner that had stressed about the appearance and placement of their system’s manhole covers reflected, “Four years later I don’t really care. I painted them green and painted some [animals] on them. It’s kind of fun.” Those that confronted concerns around noise and smell were also able to modify their systems or their placement to address those concerns. A homeowner who altered the placement of their system to accommodate noise shared, “You need to be careful about this because when you have your windows [open] in the summer you don’t want to be hearing this pump roaring all night.” Another homeowner concerned about smell was able to address this by placing charcoal caps on the exhaust pipe. That these concerns could mostly be addressed allowed some homeowners to view these systems in a more positive light, which has implications for the future diffusion of these systems through these homeowners’ social networks.

### 3.5 Reflections on adoption

In describing their experience post-installation, most participants expressed being satisfied with their system. This applied to both homeowners required to upgrade as well as those that opted into I/A pilots. “In retrospect, I don’t have any regrets about the decisions that we made. Overall, we’re happy with it,” shared a homeowner mandated to upgrade their system. Others from this cohort stated, “after the fact, it is what it is. I don’t even know that it’s there other than the three big sewer plates in my yard,” “I have to say we had a good experience,” and “We’re happy with it and we’re glad that we did it.” Given the absence of financial incentives for those mandated to install an I/A system and the system modifications some homeowners underwent, that these homeowners were satisfied with their experiences was surprising. For those that willingly participated in an I/A pilot, their satisfaction was more intuitive. “I’ve got the best septic system on my lane ∦ I’m glad to have it and I’ll be the one that has the longest lasting septic system from now on,” shared one of these homeowners. When participants were asked whether they would recommend an I/A system to a friend or neighbor if they had to replace their OWTS, with a caveat around cost, the answer to this question was overwhelmingly “yes.”

### 3.6 Incentivizing I/A system adoption

The factors influencing homeowners’ willingness to adopt these systems identified in this research can act as barriers or drivers to adoption depending on whether and how they are addressed. While the need to provide homeowners better information on I/A system costs, installation logistics and processes, and operation and maintenance responsibilities is an important outcome of this study, relying solely on the provision of this type of information to incentivize I/A system adoption will prove unsuccessful (McKenzie-Mohr and Smith, 1999; Kollmuss and Agyeman, 2002). Approaches centered on information provision alone have traditionally overlooked other determinants of behavior including psychological and other contextual factors (Kollmuss and Agyeman, 2002; Wilson and Dowlatabadi, 2007): strategies designed to influence behavior must be rooted in an understanding of barriers and drivers to a behavior (McKenzie-Mohr, 2000).

By identifying and describing social and cognitive aspects of I/A system adoption previously understudied, this research contributes to the general understanding of potential barriers and drivers to I/A system adoption upon which environmental managers and decision-makers can design behavior change strategies. One such strategy, social marketing, entails identifying barriers to a desired socially or environmentally beneficial behavior and applying strategies to remove those barriers while promoting the drivers of that behavior (McKenzie-Mohr and Smith, 1999; Johnson, 2016). Drivers of I/A system adoption identified in this study include a perceived need to replace an old or failing septic system, a desire to protect the environment and more specifically water quality, and the availability of financial incentives to help mitigate the cost of adoption. If not addressed, the perceived unaffordability of these systems and a lack of access to information on I/A systems and the adoption process are among the most significant barriers to system adoption. The focus groups revealed similar results to Warner et al. (2022) who found that managers should emphasize compatibility and observability to build support for conversions from septic to sewer (technology adoption).

A myriad of different strategies could be applied within the social marketing framework to incentivize the voluntary adoption of these systems based on these considerations, including but not limited to: establishing social norms around the adoption of I/A systems; providing financial incentives for their installation; carefully framing information on these systems around considerations that make up homeowners’ mental model of I/A system adoption and around homeowners’ interests; and employing trusted communicators to share information on these systems (McKenzie-Mohr and Smith, 1999; Johnson, 2016). The confidence in I/A system technology that non-profit pilot organizations provided early adopters despite significant informational needs highlights the importance of communicating through trusted sources (McKenzie-Mohr and Smith, 1999; Johnson, 2016).

## 4 Conclusion

As the ultimate adopters of I/A systems, homeowner perspectives and experiences matter, especially where adoption is voluntary. The literature on adoption of similar technologies and the findings of this research reinforce the need to consider factors beyond an innovation’s technological and economic efficiency. As put forth in technology adoption theory, this research confirms that social processes, communication, and the perceived characteristics of an innovation determine adoption (Wilson and Dowlatabadi, 2007; Straub, 2009). While this work provides a case study of efforts on Cape Cod, this research begins to address a notable gap in the understanding of homeowner decision-making around the adoption of nutrient-mitigating technologies outside of agriculture. Insights into the conditions and considerations that homeowners perceive to be relevant when deciding to install an I/A system allow us to identify characteristics of homeowners most likely to adopt these systems on Cape Cod, and likely in other areas as well. While these homeowner characteristics do not predict adoption, the identification of these characteristics will allow for more targeted outreach by organizations encouraging adoption, which may increase the likelihood of adoption. Homeowners who believe they need to replace a septic system or cesspool because it is old or failing or who must comply with local environmental regulations were the earliest adopters. Other characteristics of adopters included receiving financial incentives to install an I/A system, having access to their own money and being willing to spend it, and caring about local water quality and the environment.

Among the perceived characteristics of I/A systems and their installation that influenced homeowners to adopt them was the availability of accessible and trustworthy information on these systems; their affordability; and the anticipated appearance, noise, and smell associated with these systems. Affordability emerged as the most prominent inhibitor of adoption, and the use of financial incentives as a tool to mitigate perceived unaffordability cannot be understated. Uncertainty manifested itself as an underlying theme that thread through nearly every aspect of adoption, including cost, how the I/A system technology functioned, homeowners’ maintenance and monitoring responsibilities, and system aesthetics. This uncertainty could perhaps be quelled through informational campaigns targeted at these sources of uncertainty, though that information alone will not lead to behavior change.

When these systems are no longer being piloted, the financial incentives that ultimately enabled most of our participants to adopt I/As may disappear. Without these incentives, will widespread adoption of I/A systems be feasible? Based on the experiences of our study participants, it is difficult to see this happening without a change in regulations to mandate adoption, and sustained financing. A number of study participants could afford to establish themselves on the water, in wealthier communities, and build or modify their homes, and the cost burden was still onerous for this group even after financial incentives were allotted. This indicates potential challenges for adoption in other areas that face higher economic burdens.

To encourage the adoption of I/A systems among homeowners who are less willing, behavior change strategies can be applied to reduce barriers to adoption. By identifying conditions and considerations that both facilitate and inhibit adoption, this study is an important first step as successful strategies that influence behavior must be rooted in the understanding of barriers (or drivers) to a desired action (McKenzie-Mohr, 2000). If these systems are to be more widely adopted to achieve water quality goals in Cape Cod, and by extension, in other regions seeking to use these systems to address nutrient loading, improvements in communicating how I/A systems work, including the installation process and the accessibility of information on these systems, will need to be prioritized. Future case studies on how behavior change strategies such as social marketing could be applied using these focus group findings would further inform how to incentivize the adoption of these systems. Being upfront about the benefits and drawbacks of these systems, especially newer generations that are still in piloting phases, will be important. This more nuanced understanding of the factors influencing homeowners’ decision to adopt I/A systems will allow for more targeted communication and outreach regarding these systems and their installation, reducing uncertainty and ultimately guiding the design of interventions aimed at encouraging adoption. If these systems are to be used to achieve water quality goals, simply waiting for people to age out of cesspools and traditional septic systems will not suffice.

## Figures and Tables

**TABLE 1:** Individual interview and focus group discussion questions.

One-on-One Interview Questions	□ Were there any statements [from the Q sort] that really stood out to you? Why? □ Were there any statements that you found confusing? □ Were there any statements that were particularly hard to sort? □ Were there any factors or topics you considered when deciding whether to install an Innovative/Alternative (I/A) septic system that were not mentioned in these cards? □ Can you estimate how much your I/A system cost you in total? About how much of that do you estimate went to landscaping? * *Asked of adopters in focus groups 2 & 5 only
General Group Discussion Questions	□ What were your biggest motivations for installing one of these systems [probe: why did you choose to install the system? Did anyone do it because their system was really old, to add a bedroom, for financial incentives … ]?* □ What did you perceive as the biggest risk when initially considering the installation of an I/A system? □ What was your experience like before, during, after installation?** □ Is there anything you know now about I/A systems, or about the process of installation, that you wish you knew prior to installation?** □ Do you think I/A septic systems are/could be beneficial for your community? Why or why not? □ Has maintenance and monitoring been complicated?** □ Have any of you met with a contractor or engineer yet? If so, what has your experience been like so far?**** □ Did you do research on different systems when you chose what to put in? Did you find it difficult to find information on them? □ Are the long-term costs of owning and operating your I/A system what you expected?** □ If a friend or neighbor had to upgrade their septic system, would you recommend them an I/A system? □ Is there anything you’d like to add or share about your system or your installation experience (*participants in focus group 3 (non-adopters) were asked “is there anything you’d like to add or share about I/A systems”)*? □ What do you think would be a reasonable incentive to encourage people to do this in the future? *** * Prospective adopters asked “what are your biggest motivations for wanting to install an I/A septic system?” ** Asked to adopters only, in focus groups 1,2,5 *** Asked if there was sufficient time left **** *Asked to prospective adopters only, in focus group 4*

This is the semi-structured interview instrument used in the focus groups following the Q-sort activity.

**TABLE 2:** Homeowners’ informational needs before, during, and after installation.

Type of Informational Need	Description
Cost	•Cost of installing I/A system •Cost of engineering, landscaping •Ongoing costs: maintenance, monitoring, month-to-month operational cost
Operation, Monitoring, Maintenance	•How I/A systems work •I/A system longevity compared to standard septic •Whether a system will work as promised in 8, 10 years •What happens if the system stops working •Homeowners’ maintenance and monitoring responsibilities over time •Electrical needs of I/A systems •For sawdust and woodchip-based systems, the lifespan of these mediums, their ability to remove N overtime, and how to replace these mediums
Installation Process	•Positioning of the system •Space requirements for the system, and smaller I/A system options or the potential for modification where space is limited •Duration of installation
Aesthetics, Noise, Smell	•What will be underneath the ground •What the system will look like in the yard •How anticipated noise and smell might impact system siting
Other	•Whether system must be accessible 24/7 •Whether better alternatives to I/A systems will emerge in future •Whether having an I/A system will influence home resale value

## Data Availability

The datasets presented in this study can be found in online repositories. The names of the repository/repositories and accession number(s) can be found below: Supplemental materials including the focus group guide; focus group instructions sent to participants; Q-sort statements, individual interview questions, and semi-structured discussion questions; focus group recruitment letter; and the screener for recruiting participants can be found on zenodo.org, DOI 10.5281/zenodo.6264266.

## Footnotes

1 https://www.capecodcommission.org/our-work/wastewater/.

2
https://www.reclaimourwater.info/SepticImprovementProgram.aspx

3
https://www.epa.gov/water-research/innovativealternative-septic-systems

4
https://www.mass.gov/doc/310-cmr-15000-proposed-revisions-clean/download

5
https://www.epa.gov/septic/septic-systems-case-studies-and-demonstration-projects

6
https://zenodo.org/record/6264266#.Y0hABXbMJPY
